# Thyroid Hormone Replacement in Patients Following Thyroidectomy for Thyroid Cancer

**DOI:** 10.5041/RMMJ.10229

**Published:** 2016-01-28

**Authors:** Zeina C. Hannoush, Roy E. Weiss

**Affiliations:** Division of Endocrinology, Diabetes and Metabolism, Department of Medicine, University of Miami, Miller School of Medicine, Miami, FL, USA

**Keywords:** Hypothyroidism, thyroid cancer, thyroid hormone, thyroid replacement, thyroid therapy, thyroidectomy

## Abstract

Thyroid hormone replacement therapy in patients following thyroidectomy for thyroid cancer, although a potentially straightforward clinical problem, can present the clinician and patient with a variety of challenges. Most often the problems are related to the dose and preparation of thyroid hormone (TH) to use. Some patients feel less well following thyroidectomy and/or radioiodine ablation than they did before their diagnosis. We present evidence that levothyroxine (L-T4) is the preparation of choice, and keeping the thyroid-stimulating hormone (TSH) between detectable and 0.1 mU/L should be the standard of care in most cases. In unusual circumstances, when the patient remains clinically hypothyroid despite a suppressed TSH, we acknowledge there may be as yet unidentified factors influencing the body’s response to TH, and individualized therapy may be necessary in such patients.

## INTRODUCTION

### Overview

Thyroid hormone replacement (THR) therapy in athyreotic patients following thyroidectomy for thyroid cancer should be a relatively straightforward clinical problem to solve. The thyroid is absent, the hormone levels are low, the physician prescribes a dose of levothyroxine to replace what the body would otherwise manufacture, and that resolves the problem. Synthetic levothyroxine (L-T4) has been available since the 1960s, and despite its availability for 60 years physicians can be divided in their treatment of these patients.^1^ Standard of care is to titrate the L-T4 to suppress the serum thyroid-stimulating hormone (TSH) level, which is otherwise trophic for growth of normal as well as malignant thyroid cells.^2^–^7^ Nevertheless, as simple a clinical intervention as this seems, one of the most common dissatisfactions for athyreotic patients is the perception that the dose of thyroid hormone (TH) is incorrect, resulting in complaints of lethargy, weight gain, fatigue, and “brain fog.”^8^,^9^ In fact some patients hesitate to have a thyroidectomy for thyroid cancer because of the widespread perception that all patients following surgery end up with the problems above. While most patients do well on THR with serum TSHs in the range of 0.01 to 0.3 mU/L, a proportion of patients requesting multiple referrals to endocrinologists or naturopathic practitioners are dissatisfied with their treatment. The aim of this paper is to review the basis for rational THR and to identify the main pitfalls encountered in patients following thyroidectomy for thyroid cancer.

### Physiology of Thyroid Hormone

Thyroid hormone plays a critical role in the development and function of virtually every organ system in humans.^10^,^11^ This process is stimulated by TSH. The anterior pituitary releases TSH in response to thyroid-releasing hormone, which is secreted by the hypothalamus. The thyroid gland secretes both thyroxine (T4) and triiodothyronine (T3), which exert a negative feedback on TSH releasing hormone and TSH secretion.^10^,^12^

The thyroid gland secretes mainly T4, which is deiodinated intracellularly to the active hormone T3 which then binds to thyroid hormone receptors and functions as a transcription factor for many cellular processes. In the absence of a thyroid gland, exogenous L-T4 is efficiently converted to T3. Serum T3 levels remain stable after L-T4 administration but vary widely after oral administration of liothyronine (L-T3). Moreover, L-T4 is a better regulator of TSH secretion as it is more likely than T3 to pass the blood–brain barrier.^13^ This is why L-T4 has been the drug of choice for long-term treatment of athyreotic individuals.^14^

### Considerations of the Pharmacodynamics and Pharmacokinetics of Thyroid Hormone

More than 99% of circulating thyroid hormones are bound to plasma proteins, thyroid-binding globulin (TBG), T4-binding prealbumin (transthyretin), and albumin. Only the unbound or free thyroid hormone concentration is metabolically active.^12^

There are many factors that can interfere with L-T4 bioavailability and efficacy that need to be taken into consideration when prescribing THR therapy. The adequacy of L-T4 replacement dose depends, among other things, on the patient’s age, sex, menopausal status, and body weight.^15^ Several substances are known to interfere with intestinal absorption of L-T4, such as cholestyramine, aluminum hydroxide, calcium carbonate, ferrous sulfate, and sucralfate. Other substances can increase the hepatic metabolism of L-T4 (e.g. carbamazepine, phenytoin, and phenobarbital),^16^ whereas estrogens such as oral contraceptives can increase TH requirements by increasing serum levels of T4-binding globulin.^17^

The half-life of L-T4 in a euthyroid patient is approximately 7 days. In order to obtain a biological and pharmacologic steady state, adequacy of L-T4 therapy should be verified by measuring serum TSH levels no more frequently than every 4–8 weeks after the start of therapy. There is no indication for routine measurement of free T4 concentrations in patients on L-T4 when TSH is being monitored, unless the physician is concerned that the patient is not compliant with the prescribed dose. If T4 measurements are drawn, it should be after an overnight fast and before the daily dose of L-T4.^14^

A proper understanding of the pharmacokinetics of thyroid hormones is essential for treatment planning. When T4 is taken orally, up to 80% of it is absorbed. Its peak serum concentration is reached 2–4 hours after ingestion and, with the half-life noted above, a single daily dose is sufficient. As food intake reduces L-T4 absorption, patients should be advised to take their medication on an empty stomach.^14^,^18^

On the other hand, the absorption of T3 is around 90%, peak levels are reached 1–2 hours after ingestion, the serum concentration may rise by 250% to 600%, and it has a shorter half-life of about 19 hours. Because of these characteristics, L-T3 administration results in widely variable serum levels, making it a less appropriate form of thyroid hormone replacement.^14^,^18^

## FORMS OF THYROID HORMONE REPLACEMENT

### Brand versus Generic

Several different branded and generic formulations of L-T4 are commercially available (e.g. Euthirox, Synthroid, and Tirosint). Multiple studies have been conducted evaluating the bioequivalence of these different thyroid hormone formulations. Dong et al. compared four generic and brand name L-T4 preparations and found that they were bioequivalent by Food and Drug Administration (FDA) criteria and interchangeable in the majority of patients.^19^ However, the FDA’s method of determining bioequivalence of these formulations has been consistently criticized by professional societies, including Endocrine Society, American Association of Clinical Endocrinologists, and American Thyroid Association (ATA).^12^,^20^ Based on the concern that products judged to be bioequivalent do not have therapeutic equivalence and that switching products could lead to perturbations in serum TSH levels, the ATA recommends avoiding switches between levothyroxine products.^11^ Ideally, exogenous L-T4 preparations should be close to 100% pure, with less than 3% variation in L-T4 content. To ensure optimal dosing, each patient should always receive the same preparation, and, if changes need to be made for any reason, serum TSH levels should be closely monitored. This is of particular concern in frail patients, those with thyroid cancer, and the pediatric age group.^21^

### Tirosint

Recently, oral soft gelatin capsules (Tirosint, L-T4 sodium capsules), containing L-T4 dissolved in gelatin, glycerin, and water, have become available. Evidence from retrospective studies suggests that these preparations may have a more favorable absorption profile compared to standard L-T4 tablets.^22^ These capsules have a potential utility in patients affected by changes in gastric pH such as those with chronic gastritis or lactose intolerance or those receiving histamine H2 receptor blockers and proton pump inhibitors. These liquid L-T4 preparations may allow 100% absorption from the gastrointestinal tract with relatively immediate dissolution apparently unaffected by pH.^23^ Furthermore, in the rare instance when patients are intolerant of standard L-T4 preparations due to dye or other compounding materials, Tirosint formulation may be better tolerated.

### Thyroid Extracts

Desiccated thyroid or thyroid extract refers to preparations that are derived from the thyroid gland of domesticated animals. The most commonly used form of desiccated thyroid, known as Armour Thyroid, is of porcine origin and can be viewed as a mixture of T3 and T4.^24^ The main concern with the use of these preparations is centered on their T3 component. The ratio of T4 to T3 in desiccated thyroid preparations is 4.2:1, which is significantly lower than the 14:1 ratio of secretion by the human thyroid gland. This relative excess of T3 leads to supraphysiological levels of T3. In addition, due to the shorter half-life of T3, fluctuations of T3 occur over the course of the day, with peak levels shortly after dosing. Thus there is concern for thyrotoxicosis if thyroid extract therapy is not adjusted according to the serum TSH.^25^ These high T3 levels may be of particular concern in patients receiving suppressive therapy for thyroid cancer using a thyroid extract.^11^

### Liothyronine

L-T3 tablets are commercially available in the United States as Cytomel. Therapy with synthetic L-T3 has the theoretical advantage of bypassing the T4-to-T3 conversion step; however, the ability of the body’s tissues to use this T3 is not clear, and therefore there is no physiologic basis for its use. Futhermore, direct treatment with L-T3 also has the disadvantage of not permitting regulated time-specific and tissue-specific production of T3 through peripheral deiodination of T4. With T3 monotherapy, multiple daily dosing is required to sustain serum T3 levels due to the shorter half-life of L-T3 compared to L-T4.^26^

Jonklaas et al. showed that normal T3 levels can be achieved with traditional L-T4 therapy alone in patients who had undergone near-total or total thyroidectomy, which suggests that T3 administration is not necessary to maintain serum T3 values at their endogenous pre-thyroidectomy levels.^27^

If normalization of serum TSH is the goal of L-T3 monotherapy, serum T3 levels must be significantly higher than (approximately double) those seen during L-T4 monotherapy; whether this leads to relative hyperthyroidism in some tissues requires further study.^11^ Because of the widely varying serum levels that result from T3 administration, its use is no longer considered advisable. It is used only on a short-term basis in preparation for radioiodine administration.^14^,^18^

## SUPPRESSION OF TSH IN THYROID CANCER

Thyroid hormone replacement therapy after thyroidectomy in thyroid cancer is not only required to replace endogenous TH, but it is generally thought to inhibit tumor growth indirectly by its negative feedback effects on pituitary TSH secretion.^4^ Differentiated thyroid cancer expresses the TSH receptor on the cell membrane and responds to TSH stimulation by increasing the expression of several thyroid-specific proteins (thyroglobulin, sodium-iodine symporter) and by increasing the rates of cell growth.^4^,^28^ Suppression of TSH, using supraphysiological doses of L-T4, is used commonly to treat patients with thyroid cancer in an effort to decrease the risk of recurrence.^2^–^7^

The rationale for administering L-T4 therapy to patients with papillary or follicular thyroid carcinomas is based on evidence collected in numerous studies.^6^ Pujol et al.^29^ showed that thyroid cancer patients with TSH levels that were consistently below 0.1 mU/L had an improved rate of relapse-free survival compared with those whose TSH levels were always above 1.0 mU/L, and this effect was independent of age, gender, histology, and tumor stage. However, these studies did not differentiate between the beneficial effects of TSH suppression according to the aggressiveness of differentiated thyroid cancer.

Cooper et al.^4^ showed that a lesser degree of TSH suppression was an independent predictor of progression in patients with high-risk (stage III or IV) papillary thyroid cancers, but not in those with low-risk tumors. Later studies have obtained similar results.^5^,^30^ However, no further improvement in survival was found when TSH suppression was lower than 0. 1 mU/L.^31^

These results suggest that TSH suppression therapy decreases the rate of recurrences and mortality only in patients with high-risk differentiated thyroid cancer.^12^ Although the reduction in serum levels of thyroglobulin associated with low TSH levels provide indirect evidence of the efficacy of TSH suppression, increasing the degree of suppression to produce serum TSH levels below 0.5 mU/L does not result in further decrease in thyroglobulin levels. This supports the hypothesis that more aggressive TSH suppression might be of little benefit in terms of limiting tumor growth.^32^

Based on the evidence available, the ATA Management Guidelines for Patients with Thyroid Nodules and Differentiated Thyroid Cancer^33^ recommends initial TSH suppression to below 0.1 mU/L for high-risk and intermediate-risk thyroid cancer patients, while maintenance of the TSH at or slightly below the lower limit of normal (0.1–0.5 mU/L) is considered appropriate for low-risk patients.

The role of TSH suppression during thyroid hormone therapy in the long-term follow-up of patients with differentiated thyroid cancer is an area of great uncertainty as few data exist on the risk of recurrence and death from thyroid cancer associated with varying serum TSH levels at 6–12 months post-surgery and radioactive iodine ablation. Current ATA guidelines recommend maintaining TSH levels below 0.1 mU/L in patients with persistent disease indefinitely in the absence of specific contraindications. In patients with a biochemically incomplete response to therapy the serum TSH should be maintained between 0.1 and 0.5 mU/L, taking into consideration the initial ATA risk classification. In patients who are clinically and biochemically free of disease, but who presented with high-risk disease, consideration should be given to maintaining TSH suppression with TSH levels of 0.1–0.5 mU/L for 5 years. In patients free of disease and with low risk for recurrence the serum TSH may be kept within the low normal range (0.5–2 mU/L), and, finally, in patients who have not undergone remnant ablation, who are clinically free of disease, and have undetectable suppressed serum thyroglobulin levels and normal neck ultrasound, the serum TSH may also be allowed to rise to the low normal range (0.5–2 mU/L).^33^

The issue of determining which is the appropriate amount of suppression needed for each specific case is relevant because of the potential undesirable side effects of over-suppression. Adverse effects of TSH suppression may include the known consequences of subclinical thyrotoxicosis, including exacerbation of angina in patients with ischemic heart disease, increased risk for atrial fibrillation in older patients, and increased risk of osteoporosis in post-menopausal women.^34^,^35^ In addition to the potential for adverse effects on heart and bone, TSH suppression therapy is not without possible other risks in patients with differentiated thyroid cancer. An increased incidence of kidney, pancreas, ovarian, and breast cancers has been noted.^36^ It has been postulated that integrin activation by TSH could be responsible for promoting angiogenesis through the activation of MAPK (or MAPK pathway).^36^

Stratification into a risk-adapted management scheme for L-T4 therapy in patients with differentiated thyroid cancer has been proposed, considering aggressiveness of cancer, patient age, and presence of cardiovascular and skeletal risk factors.^28^ In general, long-term treatment with L-T4 should be individualized and balanced against the potential risk of adverse effects during the follow-up in patients who are both at high risk of recurrence and at high risk of adverse effects. This is particularly relevant in the elderly population who are at increased risk for both cancer progression and L-T4 therapy side effects.^12^

## SPECIAL CONSIDERATIONS

### Adjustment of THR during Pregnancy

One particularly challenging situation is thyroid hormone replacement in pregnancy, where dose adjustments are usually required. During pregnancy, the production of T4 and T3 is increased by 50%, and the daily iodine requirement is increased by 50%. The TSH normal reference range in pregnancy is influenced by high T4-binding globulin, estrogens, human chorionic gonadotropin levels, increased iodine clearance, and enhanced type 3 deiodinase activity of the placenta. Recent guidelines state that the upper limit of normal of TSH should be 2.5 mU/L in the first trimester of pregnancy and 3 mU/L in the second and third trimesters.^37^ In women receiving L-T4 for replacement alone, the dose should be increased by 30% as soon as pregnancy is confirmed. In women receiving suppressive therapy, hormone levels should be checked every month during pregnancy, and the LT4 dose is increased if serum TSH level increases. It is not well established in current guidelines whether the TSH level goal should be lowered in women with history of thyroid cancer during pregnancy. Each physician needs to make an individualized clinical judgment taking into consideration the recurrence risk of the patient. The pre-pregnancy dose of L-T4 should be immediately resumed after delivery.^14^,^38^

### THR in Pediatric Patients with Thyroid Cancer

Thyroid hormone replacement in children presents its own unique set of challenges. While children are not likely to complain of decreased energy, concerned parents may tend to transfer their perception of what thyroid hormone should do to the child’s activity level. The discussion of TSH suppression following thyroidectomy for thyroid cancer in adults also applies to children. Nevertheless, the ability to achieve consistent TSH suppression in children is difficult mainly due to higher non-compliance rates with the medication. When the home environment is not conducive to compliance, other measures may need to be taken. Anecdotally for one particularly non-compliant child, we have had the medication administered to the patient by the school nurse (and during the summer vacation a visiting nurse). It was given as a single weekly dose, and serum TSH levels were consistently kept below 0.5 mU/L. In general, weight-based requirements for thyroid hormone are increased in children and adolescents compared to adults,^39^ and for this reason TSH should be checked more frequently.

No data are available to compare the outcomes, risks, and benefits of different TSH suppression levels in children with thyroid cancer. Recognizing the limited data available, the ATA has submitted guidelines based on risk level and current disease status that recommend maintaining TSH suppression in children with known or suspected persistent disease. In children with no evidence of disease, the TSH can be normalized to the low normal range after an appropriate period of surveillance. In pre-pubertal children bone maturation and pubertal development should be monitored. Extrapolating from patients with Graves’s disease, the potential risks of TSH suppression include growth acceleration, advanced bone age, early-onset puberty, reduced bone mineral content, poor academic performance, and tachyarrhythmia.^40^

### THR in Non-thyroidal Illness Syndrome

Critically ill patients who were otherwise euthyroid prior to illness or those severely malnourished demonstrate suppressed serum T3 and often T4 and TSH, with an increase in reverse T3. This pattern is known as non-thyroidal illness syndrome or “euthyroid-sick syndrome.” It is likely due to a suppression of the hypothalamic–pituitary–thyroid axis, a downregulation of type 2 deiodinase resulting in decreased T4-to-T3 conversion, and an increase in reverse T3 production. Whether this is a physiologic adaptation or a pathologic response is a question of great controversy.^41^,^42^ The thyroid hormone status of the tissues in a critically ill patient is unknown and may not correlate with serum levels. Nevertheless in athyreotic patients who are critically ill the rational approach is to treat with T3 until the patient recovers, monitoring T3 and TSH serum levels. Once the critical illness is corrected, return to T4 replacement should occur. It should be noted that there is no clinical or experimental evidence to support the preferential use of T3 over T4 in athyreotic patients who are critically ill.

### Patients with Persistent Hypothyroid Symptoms despite a Suppressed TSH

L-T4 is the standard of care for thyroidectomized patients, is very efficient at achieving the desired TSH suppression in thyroid cancer cases, and in most patients it results in resolution of the signs and symptoms of hypothyroidism. However, a small proportion of patients (although ones that seem to find their way to multiple endocrinologists) being treated with L-T4 feel that this medication is not efficacious in restoring optimum health. One possible explanation for this phenomenon may be genetic variations in the expression of peripheral 5′-deiodinases, which are selenoprotein enzymes that catalyze the conversion of T4 to active T3 in the peripheral tissue.^43^ Patients with low 5′-deiodinase activity may be unable to metabolize T4 to T3 in adequate amounts and may therefore respond better to combined replacement therapy than to T4 alone.^44^ There is, however, no routinely available clinical biochemical or genetic test to determine whether this is the case,^18^ and each treating physician needs to weigh the unlikely occurrence of this rare possibility versus gaining the confidence of the patient that their physician is listening to them and concerned for their care. Most often a detailed discussion with the patient will reveal that they were not completely satisfied with their weight or health prior to the thyroidectomy. The presence of anti-thyroid antibodies may somehow be involved in these symptoms.

### Patients Unable to Tolerate a Suppressed TSH

Patients with a history of thyroid cancer are encouraged to keep their serum TSH at least below 0.5 mU/L (see discussion above). However, there will be those patients in whom any slight decrease in the serum TSH results in palpitations and apparent increased sensitivity to TH.^8^ Such individuals may have had pre-thyroidectomy serum TSH values at the higher part of the normal range and, due to as yet undefined molecular pathways, are unable to tolerate a higher dose of thyroid hormone. In such instances the clinician will need to weigh the risks and benefits of suppressive therapy.

### Patients Unable to Achieve a Suppressed TSH despite Proper Thyroid Hormone Therapy

There are some patients on adequate or even high doses of thyroid hormone therapy who are unable to achieve TSH suppression. The differential diagnoses include malabsorption, non-compliance, factors increasing the medication’s metabolism, or increased serum levels of T4-binding globulin. In addition, when the TSH cannot be suppressed in spite of adequate doses of thyroid hormone, the physician should consider the presence of heterophile antibodies and interference with the laboratory measurement including anti-mouse antibodies, rheumatoid factor, and autoimmune anti-TSH antibodies. Finally one could consider the coexistence of adrenal insufficiency, which may induce TSH elevation reversible with glucocorticoid replacement.^12^

Defects in thyroid hormone absorption are rare without a history of previous gut surgery, celiac disease, lactose intolerance, autoimmune gastritis, or *Helicobacter pylori* infection*.* A serum free T4 peak at 2 hours rising above the upper limit of normal after the administration of 100 μg of L-T4 suggests proper absorption, but unfortunately there are no well-established standards for this test.^45^ A radioisotope-labeled L-T4 tracer technique may be used to test absorption more accurately, but this technique is not readily available. Prior studies looking into this matter have shown that oftentimes patients suspected to have absorption problems actually exhibit a factitious disorder and have compliance issues.^45^

One daily dose of L-T4 accounts for 14% of the total weekly dose. Therefore a missed dose of L-T4 may affect free T4 and TSH levels over several weeks due to the long half-life of L-T4. The prevalence of non-compliant hypothyroid patients has been reported between 30% and 80% despite the simplicity of once-daily dosage of the medication.^46^ There are many possible causes for patient non-compliance, and special attention should be paid to try to address common psychosocial causes such as barriers to accessing the medication, difficulties with insurance coverage, literacy issues, and lack of understanding regarding the benefits of taking L-T4 as a medication.

If efforts to encourage regular daily consumption of L-T4 are unsuccessful, alternative options for medication delivery include twice weekly or once weekly dosage.^11^,^47^ However, only three crossover trials of such oral therapy have been conducted, and none of them were long-term or conducted in a patient group reported as non-adherent.^48^–^50^ The once-weekly or twice-weekly dosage approach should be avoided in patients with underlying heart disease because of the potential exacerbation of congestive heart failure and arrhythmias due to transient supraphysiological hormone concentrations achieved in the first 1 or 2 days of therapy.^12^

For patients with proven malabsorption issues, a weekly intramuscular injection of L-T4 may be a useful therapeutic approach in obtaining biochemical and clinical euthyroidism. Intravenous thyroid hormone therapy is another alternative, but it is not universally available.^51^ Because about 70% of an orally administered dose of T4 is absorbed, individuals unable to ingest L-T4 should initially receive 70% or less of their usual oral those when the medication is given intravenously. Crushed L-T4 tablets suspended in water should be given through a naso-gastric tube to patients receiving enteral feeding.

One possible rare cause of inability to achieve a suppressed TSH despite high doses of L-T4 is thyroid hormone resistance, also referred to as impaired sensitivity to thyroid hormone. This is a rare syndrome in which the thyroid hormone levels are elevated but the TSH level is not suppressed, or not completely suppressed as would be expected.^52^ However, it is very unlikely that this scenario would present *de novo* after thyroidectomy for thyroid cancer, and assessing pre-thyroidectomy thyroid function tests is of vital importance to establish normal function of the hypothalamic–pituitary–thyroid axis.

## CONCLUSION

Thyroid hormone replacement therapy in athyreotic patients with a history of thyroid cancer is a straightforward clinical problem that needs to take into account the form of TH used, the timing of the blood tests to assess TSH concentrations, and other factors that affect the absorption and/or biologic availability of this important hormone. While the majority of patients have no difficulty being maintained with THR, there will predictably be special patients who find themselves in the doctor’s office, or make contact by phone or email, with the concern that they are not being properly replaced with TH. This may not correlate with the serum TSH level and may reflect yet unidentified molecular mechanisms of thyroid hormone action that do not tell the entire story of what it takes to replace the body’s production of thyroid hormone.

## Abbreviations:

ATA: American Thyroid Association
FDA: Food and Drug Administration
L-T3: liothyronine
L-T4: levothyroxine
T3: triiodothyronine
T4: thyroxine
TBG: thyroid-binding globulin
TH: thyroid hormone
THR: thyroid hormone replacement
TSH: thyroid-stimulating hormone.
